# Two Adjacent and Similar TetR Family Transcriptional Regulator Genes, *SAV577* and *SAV576*, Co-Regulate Avermectin Production in *Streptomyces avermitilis*

**DOI:** 10.1371/journal.pone.0099224

**Published:** 2014-06-10

**Authors:** Jia Guo, Xuan Zhang, Zhi Chen, Ying Wen, Jilun Li

**Affiliations:** State Key Laboratory of Agro-Biotechnology and MOA Key Laboratory of Soil Microbiology, College of Biological Sciences, China Agricultural University, Beijing, P. R. China; Baylor College of Medicine, United States of America

## Abstract

*Streptomyces avermitilis* is an important bacterial species used for industrial production of avermectins, a family of broad-spectrum anthelmintic agents. We previously identified the protein SAV576, a TetR family transcriptional regulator (TFR), as a downregulator of avermectin biosynthesis that acts by controlling transcription of its major target gene *SAV575* (which encodes cytochrome P450/NADPH-ferrihemoprotein reductase) and *ave* genes. *SAV577*, another TFR gene, encodes a SAV577 protein that displays high amino acid homology with SAV576. In this study, we examined the effect of SAV577 on avermectin production and the relationships between SAV576 and SAV577. SAV577 downregulated avermectin biosynthesis indirectly, similarly to SAV576. SAV576 and SAV577 both directly repressed *SAV575* transcription, and reciprocally repressed each other's expression. *SAV575* transcription levels in various *S. avermitilis* strains were correlated with avermectin production levels. DNase I footprinting and electrophoretic mobility shift assays indicated that SAV576 and SAV577 compete for the same binding regions, and that DNA-binding affinity of SAV576 is much stronger than that of SAV577. GST pull-down assays revealed no direct interaction between the two proteins. Taken together, these findings suggest that SAV577 regulates avermectin production in *S. avermitilis* by a mechanism similar to that of SAV576, and that the role of SAV576 is dominant over that of SAV577. This is the first report of two adjacent and similar TFR genes that co-regulate antibiotic production in *Streptomyces.*

## Introduction

*Streptomyces* bacteria produce a wide range of bioactive secondary metabolites during their complex life cycle. These metabolites include source compounds for most known antibacterial, anticancer, anthelmintic, and immunosuppressive antibiotics [1]–[3]. The biosynthesis of these antibiotics is under the control of numerous regulatory factors, including environmental and physiological factors, pathway-specific and pleiotropic regulatory factors [4]–[10]. The avermectins, well-known antiparasitic and insecticidal agents generated by *Streptomyces avermitilis* [11], [12], are widely applied in medical, veterinary, and agricultural fields and are one of the most commercially important groups of antibiotics. However, the regulatory factors and mechanisms involved in avermectin production are poorly known. Increased understanding of these factors and mechanisms will be useful in generating antibiotic overproducers, inhibiting production of undesired antibiotics, and activating production of cryptic antibiotics.

The TetR family transcriptional regulators (TFRs) are a large family of transcriptional regulators in bacteria that help control such cellular processes as antibiotic production, multidrug resistance, amino acid metabolism, osmotic stress resistance, pathogenicity, and development [13]. More than 10,000 proteins are found in non-redundant protein databases, and most bacteria contain at least one TFR [14]. Certain *Streptomyces* species contain over 100 TFRs; this large number presumably reflects the complexity of morphological differentiation and secondary metabolism in these species. Many of the functions of TFRs in *Streptomyces* remain unknown. Complete sequencing of the *S. avermitilis* genome revealed 115 predicted TFRs [15]. Of these, SAV3818 [16] and SAV3703 (AvaR3, a γ-butyrolactone-autoregulator receptor) [17] have been described as positive regulators of avermectin production, and SAV151 [18] and SAV7471 [19] as negative regulators. Investigation of other yet-uncharacterized TFRs in *S. avermitilis* will surely increase our understanding of the complex regulatory network involved in avermectin biosynthesis.

We previously applied comparative transcriptome analysis to characterize the TFR SAV576 as an avermectin downregulator and demonstrated that SAV576 inhibits avermectin production indirectly by modulating transcription of its target gene *SAV575* and *ave* genes [20]. *SAV577* is another TFR gene downstream of *SAV576*. The deduced sequence of SAV577 protein is very similar to that of SAV576 protein. Investigation of SAV577 function in the present study shows that SAV577 represses avermectin biosynthesis similarly to SAV576 and suggests that the role of SAV576 in *S. avermitilis* is dominant over that of SAV577.

## Materials and Methods

### Strains, Plasmids, and Growth Conditions

*S. avermitilis* wild-type strain ATCC31267 grown at 28°C was used for gene propagation and gene disruption. Solid YMS [21] and RM14 media [22] were used for sporulation and protoplast regeneration, respectively. Liquid YEME medium [23] containing 25% sucrose was used to grow mycelia for protoplast preparation. Seed medium and fermentation medium FM-1 used for avermectin production were as described previously [24]. Because FM-I contains insoluble yeast meal, soluble FM-II [25] was used to grow mycelia for growth analysis, RNA isolation and ChIP assay.

*E. coli* JM109 and BL21 (DE3) (Novagen, Germany) were used as cloning host and expression host, respectively. *E. coli* ET12567 (*dam dcm hsdS*) was used to propagate non-methylated DNA for transformation into *Streptomyces* [22].

*E. coli-Streptomyces* multiple-copy shuttle vector pKC1139 was used for gene disruption and overexpression in *Streptomyces* strains. pSET152 was used to integrate a single-copy gene into the *Streptomyces* chromosome [26]. pET-28a (+) (Novagen) was used for production of recombinant His_6_-tagged protein in *E. coli*. pGEX-4T-1 (GE Healthcare, USA) was used for expression of GST fusion protein in *E. coli*.

### Gene Disruption, Complementation, and Overexpression

**Figure 1:**
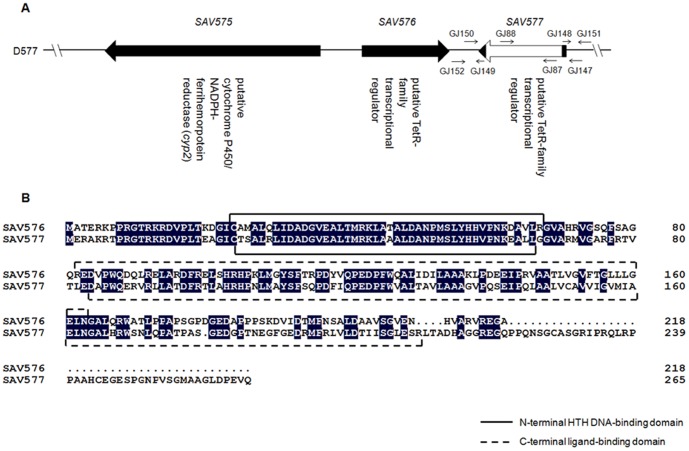
Organization of *SAV577* and its adjacent genes on the chromosome of *S. avermitilis* (A) and amino acid alignment of SAV576 and SAV577 (B). (A) Genetic organization of *SAV575*, *SAV576*, and *SAV577* genes, and the strategy for deletion of *SAV577*. Long black arrows: genes and their directions. Short arrows: positions of primers used for cloning exchange regions and confirming gene deletions. White block: in-frame deletion in *SAV577* gene. (B) Black background: identical amino acid residues.

To construct a *SAV577* deletion mutant, two fragments flanking *SAV577* were prepared by PCR from genomic DNA of ATCC31267. A 663-bp 5′ flanking region (from positions −644 to +19 relative to the *SAV577* start codon) was amplified with primers GJ147 and GJ148, and a 686-bp 3′ flanking region (from positions +787 to +1472) was amplified with primers GJ149 and GJ150. The two PCR fragments were cloned into pKC1139 to generate *SAV577*-deletion vector pDGJ577, which was transformed into ATCC31267 protoplasts. Double-crossover recombination strains were selected as described previously [27]. The resulting putative *SAV577* deletion mutants were confirmed by PCR analysis using primers GJ151, GJ152, GJ87 and GJ88 (Fig. 1A), and this was followed by DNA sequencing. When using primers GJ151 and GJ152, which flank the exchange regions, a 1.46-kb band was observed, whereas a 2.23-kb band was produced from ATCC31267 genomic DNA. When using primers GJ87 and GJ88, located within the deletion region of *SAV577*, only ATCC31267 produced a 429-bp PCR fragment as predicted (data not shown). The obtained *SAV577* deletion mutant was designated D577. For complementation of D577, a 1.4-kb DNA fragment carrying the *SAV577* open reading frame (ORF) and its putative promoter was amplified by PCR from ATCC31267 genomic DNA with primers GJ223 and GJ224. The PCR product was excised with *Eco*RI/*Xba*I and inserted into the corresponding sites of pSET152 to generate *SAV577* gene complementation vector pSET152-577, which was then introduced into D577 to obtain the complemented strain. The 1.4-kb *Eco*RI/*Xba*I fragment containing the *SAV577* gene from pSET152-577 was cloned into the corresponding sites of pKC1139 to produce pKC1139-577, which was used for overexpression of *SAV577* in ATCC31267.

To construct a *SAV576-SAV577* double deletion mutant, the *SAV576* deletion mutant D576 [20] was transformed with *SAV577*-deletion vector pDGJ577. The expected mutant, designated D576-577, was isolated using the strategy described above for the selection of *SAV577* deletion mutant D577, and was confirmed by PCR analysis using primers GJ87, GJ88, GJ151 and GJ152+ (flanking 5′ exchange region of *SAV576*). D576-577 produced no PCR product with primers GJ87 and GJ88. When using primers GJ151 and GJ152+, a 1.26-kb band was observed from D576-577, whereas a 2.64-kb band was produced from ATCC31267. All the primers used in this study are listed in Table S1.

### Real-time RT-PCR Analyses

RNAs were extracted using Trizol reagent (Tiangen, China) from *S. avermitilis* cultures grown in FM-II at various incubation times. The quality and quantity of RNAs were assessed using a NanoVue Plus spectrophotometer (GE Healthcare) and checked by electrophoresis. RNA samples were treated with DNase I (TaKaRa, Japan) to remove chromosomal DNA contamination. Each treated RNA sample (2 µg) was reverse transcribed by mixing M-MLV (RNase H^−^; TaKaRa), random hexamers (25 µM), and a dNTP mixture (10 mM each). Real-time RT-PCR analyses were performed to determine the transcription levels of various genes, using the obtained cDNA as template and the primers listed in Table S1. The *hrdB* gene, which encodes the major sigma factor in *Streptomyces*, was used as a positive internal control. The RNA sample that was not reverse transcribed was used as a negative control to rule out chromosomal DNA contamination. The experiments were performed using FastStart Universal SYBR Green Master (ROX) (Roche, USA) with analysis by an ABI 7900HT Sequence Detection System. Template cDNA, 10 µl FastStart Universal SYBR Green Master (ROX), and forward and reverse primers (each 300 nM) were mixed in each reaction system (20 µl). PCR protocol: 95°C for 10 min, 40 cycles of 95°C for 10 s/ 60°C for 30 s.

### Overexpression and Purification of the Recombinant His_6_-tagged SAV577 Protein

The *SAV577* coding region for 265 amino acids was obtained by PCR using primers GJ229 and GJ230 (Table S1). The PCR fragment was excised with *Nde*I/*Eco*RI and inserted between the corresponding sites in the expression vector pET-28a(+) to generate pET28-577, which was confirmed by DNA sequencing and then introduced into *E. coli* BL21 (DE3) for protein overexpression. The recombinant SAV577 protein tagged with His_6_ at the N-terminus was induced by IPTG and purified on a Ni^2+^-NTA spin column (Qiagen, Germany) according to the manufacturer's instructions. The purified protein was used for EMSA and DNase I footprinting assays.

### GST Pull-down Assay

To prepare GST-tagged SAV576 protein, primers GJ231 and GJ232 (Table S1) were used to amplify an 818-bp DNA fragment containing the *SAV576* coding region. To prepare GST-tagged SAV577 protein, primers GJ233 and GJ234 were used to amplify the 883-bp *SAV577* coding region. The PCR products were cloned into the expression vector pGEX-4T-1 to generate pGEX-576 and pGEX-577, respectively, which were then introduced into *E. coli* BL21 (DE3)/pET28-576 [20]. pGEX-577 was also introduced into BL21 (DE3)/pET28-577. *E. coli* BL21 (DE3) containing pET28-576 and pGEX-4T-1 was used as a negative control. Following IPTG induction, bacteria that contained both GST- and His_6_-tagged proteins were collected and resuspended in lysis buffer [20 mM Tris-Cl (pH 8.0), 200 mM NaCl, 1 mM EDTA (pH 8.0), 0.5% NP-40]. The total protein in the supernatant was recovered by ultrasonication and centrifugation at 4°C. Proteins from each cell culture were purified with glutathione-sepharose beads, and the beads were washed three times with lysis buffer. Bound proteins were eluted with SDS sample buffer, analyzed by SDS-PAGE, and subjected to Coomassie Blue staining and Western blotting with anti-His or anti-GST antibody (Tiangen).

### Western Blotting

The protein sample obtained from the GST pull-down assay was separated by 7.5% SDS-PAGE, and the separated proteins were transferred to a PVDF membrane in a Mini Trans-Blot Electrophoretic Transfer Cell at 100 mA for 2 h on ice. Western blots were developed with anti-His or anti-GST antibody using an alkaline phosphatase substrate detection system (Amresco, USA).

### Chromatin Immunoprecipitation (ChIP) Assay

ChIP assays were performed as described previously [25], but made little modification. In brief, *S. avermitilis* cultures grown in FM-II for 72 h were fixed in cross-linking buffer [0.4 M sucrose, 10 mM Tris·HCl (pH 8.0), 1 mM EDTA] containing 1% formaldehyde for 20 min at 28°C. Glycine was added (final concentration 125 mM) to stop the reaction, and incubation was continued for 5 min. ChIP was performed using anti-SAV576 antibody prepared in our laboratory [20]. After DNA extraction, pellets were washed with 70% ethanol, resuspended in 50 µl TE [10 mM Tris·HCl, 1 mM EDTA (pH 8.0)], and 2 µl DNA solution was subjected to PCR using the primer sets listed in Table S1. Input sample with genomic DNA (prior to immunoprecipitation) as template for PCR was used as the positive control. The immunoprecipitated DNA with rabbit preimmune serum was used as the negative control.

### Electrophoretic Mobility Shift Assay (EMSA)

EMSA was performed using a DIG Gel Shift Kit, 2^nd^ Generation (Roche) according to the manufacturer's instructions. The probes (listed in Table S1) were amplified by PCR and labeled with digoxigenin (DIG) at the 3′-terminal end. The reaction mixture (20 µl) contained the probes, proteins, and 1 µg poly [d (I-C)] (vial 9) in the binding buffer (vial 5). The mixture was incubated at 25°C for 30 min and then added with 5 µl loading buffer with bromophenol blue (vial 13). Protein-DNA complex and free DNA were separated by electrophoresis on native 5% polyacrylamide gels with 0.5×TBE as running buffer and transferred onto nylon membranes by electroblotting. The membranes were baked at 80°C for 10 min, and the DNA fragments were cross-linked by exposure to 254-nm UV radiation for 10 min. Chemiluminescence detection was performed according to the manufacturer's instructions, and the membranes were exposed to X-ray film (Fuji, China) for 15–30 min.

### DNase I Footprinting Assay

This assay was performed using an FAM fluorescence labeling capillary electrophoresis method [28]. Two fluorescence-labeled DNA fragments amplified with primer pairs FAM-GJ78/GJ77 and FAM-GJ228/GJ227 (Table S1) were used to characterize the binding sites of SAV577 protein in the SAV575-SAV576 intergenic region. The resulting 547-bp and 478-bp DNA fragments covered the entire intergenic region. The labeled DNA fragments (400 ng) and corresponding concentrations of His_6_-tagged SAV577 protein were added to a reaction mixture (final volume 50 µl) and incubated for 30 min at 25°C in binding buffer [20 mM HEPES (pH 7.6), 10 mM (NH_4_)_2_SO_4_, 1 mM DTT, 0.2% Tween-20, 30 mM KCl]. DNase I (0.016 units) digestion was performed for 40 s at 37°C and stopped by addition of EDTA at a final concentration of 60 mM. The reaction mixture was heated to 80°C for 10 min to totally inactivate DNase I. Samples were subjected to phenol-chloroform extraction, ethanol precipitation, and capillary electrophoresis by loading into an 3730 DNA Genetic Analyzer with the internal-lane size standard ROX-500 (Applied Biosystems, USA). Electrophoregrams were analyzed using the GeneMarker program, v1.8 (Applied Biosystems).

### Fermentation and HPLC Analysis of Avermectin Production

Fermentation of *S. avermitilis* ATCC31267 and its mutant strains was performed, and avermectins in the fermentation culture were identified by HPLC analysis as described previously [24].

## Results

### SAV577 Plays a Negative Regulatory Role in Avermectin Production

*SAV577* is located downstream of *SAV576* and transcribed divergently with *SAV576* (Fig. 1A). *SAV577* encodes a 265-amino-acid protein with an N-terminal TetR-family helix-turn-helix (HTH) DNA binding domain (Fig. 1B). The deduced SAV577 protein displays 59% amino acid identity with the SAV576 protein and has a stretch of 47 amino acid residues at its C-terminal region that are not present in SAV576. The *SAV576* and *SAV577* genes are specific to *S. avermitilis* and have no orthologs in other sequenced *Streptomyces* genomes.

**Figure 2:**
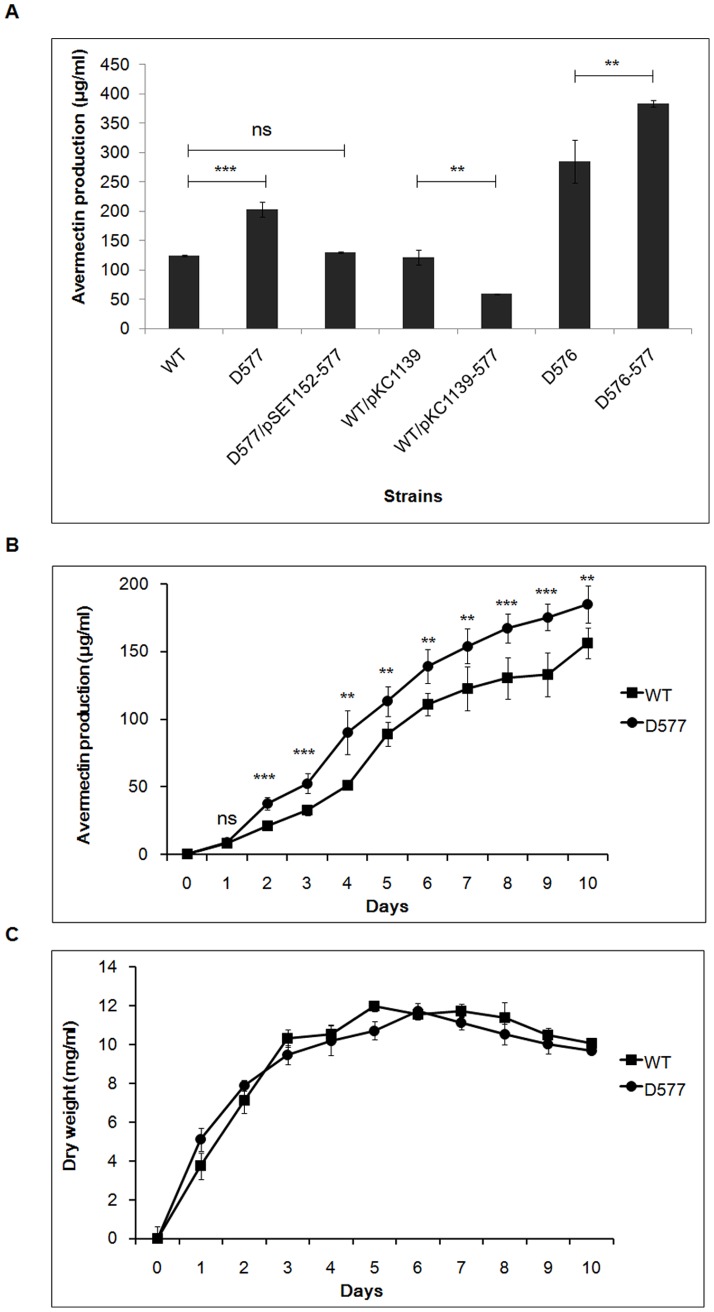
Avermectin production and growth of wild-type strain ATCC31267 and *SAV577* mutant strains. (**A**) Comparison of avermectin production in various *S. avermitilis* strains grown in FM-I medium for 10 days. WT, wild-type strain ATCC31267. WT/pKC1139, ATCC31267 carrying control plasmid pKC1139. D576, *SAV576* deletion mutant. D577, *SAV577* deletion mutant. D577/pSET152-577, complementation strain of D577. WT/pKC1139-577, *SAV577* overexpression strain. D576-577, *SAV576-SAV577* double deletion mutant. (**B** and **C**) Effect of *SAV577* deletion on avermectin production (**B**) and growth (**C**) of *S. avermitilis* grown in soluble medium FM-II. Solid squares, ATCC31267; Solid circles, D577. Results are shown as mean ± SD for three independent experiments. **P<0.01 and ***P<0.001 as determined by Student's *t*-test. ns, not significant.

SAV576 has been identified as an avermectin downregulator [20]. In view of the similarity of TFRs SAV577 and SAV576, we investigated the possible role of SAV577 in avermectin production. Deletion of *SAV577* in both wild-type strain ATCC31267 (D577) and *SAV576* deletion mutant D576 (D576-577) resulted in increased avermectin production (Fig. 2A). To confirm that this increase was due solely to *SAV577* deletion, we constructed an *SAV577* gene complementation strain (D577/pSET152-577) using a pSET152-based vector pSET152-577 that contained the *SAV577* coding region and its putative promoter. Avermectin production was restored in this complementation strain, confirming that the absence of *SAV577* was the cause of enhanced avermectin production in D577. Enhancement of *SAV577* expression by introduction of the multi-copy plasmid pKC1139-577 into ATCC31267 (WT/pKC1139-577) caused a 52.6% reduction in avermectin yield. The deletion and overexpression of *SAV577* were confirmed by real-time RT-PCR analysis (Fig. S1). In addition, *SAV577* was overexpressed in D576 relative to ATCC31267, and *SAV576* was overexpressed in D577 but underexpressed in WT/pKC1139-577 (Fig. S1), suggesting that SAV576 and SAV577 inhibit each other's expression. Taken together, these findings indicate that SAV577 has a negative role similar to that of SAV576 in regulating avermectin production and that *SAV576-SAV577* double deletion produces an additive enhancing effect on avermectin yield.

To investigate whether the avermectin overproduction of D577 was due to increased cell growth, we analyzed the growth and avermectin production of ATCC31267 and D577 cultured in soluble medium FM-II. The deletion of *SAV577* resulted in increased avermectin production (Fig. 2B), but did not affect cell growth (Fig. 2C). These findings indicate that *SAV577* inhibits avermectin biosynthesis, but has no effect on cell growth.

### *SAV577* is a Target Gene of SAV576

We demonstrated previously that SAV576 represses transcription of its own gene and the adjacent *SAV575* gene through binding to the 15-bp palindromic sequence CCRTACRVYGTATGS (R: A or G; V: A, G, or C; Y: T or C; S: G or C) [20]. Sequence analysis revealed a similar palindromic sequence (CCGTACGTAGTCCGC) within the *SAV577* promoter region, suggesting that *SAV577* is a potential target gene of SAV576.

**Figure 3:**
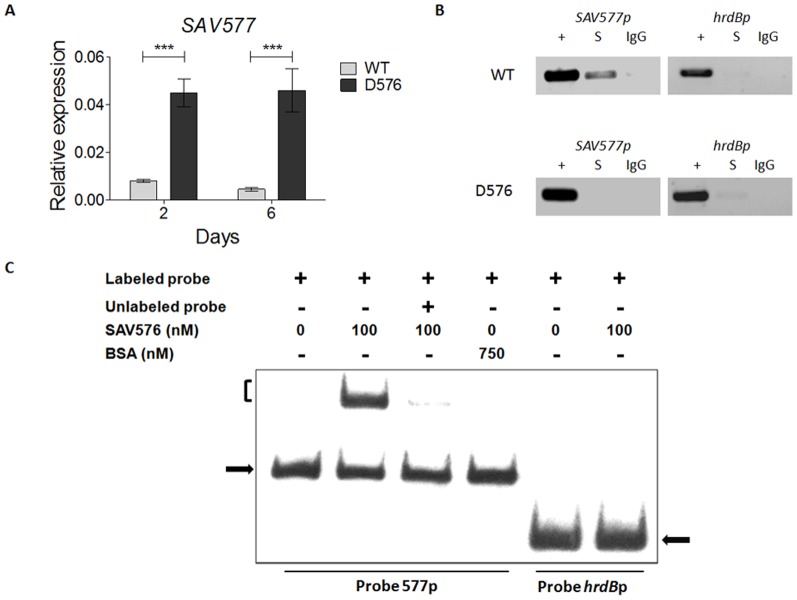
Identification of *SAV577* as a target gene of SAV576. (**A**) Real-time RT-PCR analysis of *SAV577* transcription levels for ATCC31267 (WT) and *SAV576* deletion mutant D576 grown in FM-II on days 2 and 6. Relative values were obtained using *hrdB* as a reference. Error bars, standard deviations (*n* = 3). ***P<0.001 as determined by Student's *t*-test. (**B**) ChIP assays. Anti-SAV576 antibody was used to immunoprecipitate SAV576-DNA complexes from ATCC31267 and D576 cells treated with formaldehyde. The DNAs used for PCR were total DNA prior to immunoprecipitation (positive control: lanes “+”), immunoprecipitated DNA with anti-SAV576 antibody (experimental sample: lanes “S”), and immunoprecipitated DNA with rabbit preimmune serum (negative control: lanes “IgG”). The *hrdB* promoter region was used as a control. (**C**) EMSAs of the interaction of probe 577p with purified His_6_-SAV576 protein. Each lane contained 0.3 nM labeled probe. An approximately 100-fold excess of the unlabeled probe was used in competitive assays. BSA was used as a negative control for SAV576 protein. Labeled 262-bp *hrdB* promoter region (probe *hrdBp*) was used to eliminate non-specific binding of the SAV576 protein. The free probe is indicated by solid arrow, and the retarded DNA fragment is indicated by parentheses.

To investigate the possible regulatory effect of SAV576 on *SAV577* expression, we determined transcription levels of *SAV577* by real-time RT-PCR using RNAs isolated from ATCC31267 and D576 after 2 (early exponential phase) or 6 (stationary phase) days' growth in FM-II, corresponding to the stages of previous work [20]. *SAV577* expression was markedly upregulated in D576 for both durations (Fig. 3A), indicating that SAV576 represses *SAV577* transcription. To determine whether this repressive effect was direct, we performed *in vivo* ChIP assays and *in vitro* EMSAs.

For ChIP assays, *S. avermitilis* strains were treated with formaldehyde at 72 h to cross-link the SAV576 protein to its DNA targets. A 209-bp *SAV577* promoter region was detected by PCR using the primer pairs shown in Table S1. The correct PCR products were obtained with these primers from positive control DNA (lanes “+”) of ATCC31267 and D576, whereas no such bands were detected from negative control DNA (lanes “IgG”) (Fig. 3B). Use of anti-SAV576-immunoprecipitated DNA sample (lanes “S”) of ATCC31267 as template revealed that only the *SAV577* promoter region was selectively enriched relative to the control *hrdB* promoter. In contrast, these primers could not amplify DNA fragment from anti-SAV576-immunoprecipitated DNA (lanes “S”) of mutant strain D576 (Fig. 3B). These findings indicate that SAV576 binds specifically to the *SAV577* promoter region *in vivo*.

Direct interaction of the *SAV577* promoter region with SAV576 was confirmed by EMSAs using a full-length recombinant His_6_-SAV576 protein expressed in *E. coli* [20]. The 209-bp *SAV577* promoter region used in the ChIP assays was labeled with DIG and designated as probe 577p. The His_6_-SAV576 protein clearly retarded probe 577p (Fig. 3C). Binding specificity to SAV576 was evaluated by addition of excess unlabeled probe 577p, which competes strongly with labeled probe 577p. A labeled nonspecific DNA probe and BSA were used as negative controls. The findings indicate that SAV576 regulates *SAV577* transcription directly through binding to the *SAV577* promoter region.

### SAV577 Has a Regulatory Mechanism Similar to That of SAV576

**Figure 4:**
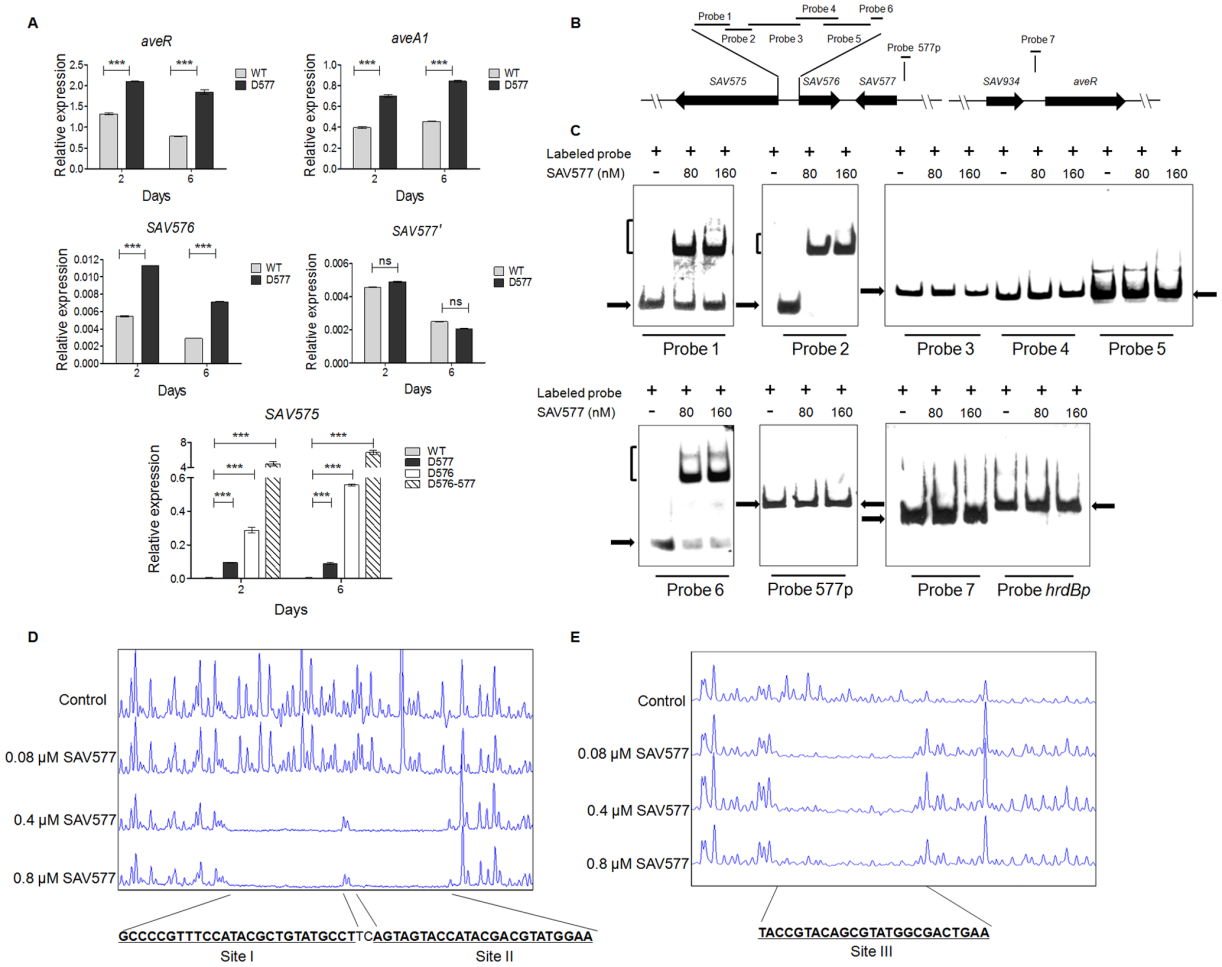
SAV577 directly represses *SAV575* and *SAV576* expression. (**A**) Effect of *SAV577* deletion on expression of *aveR*, *aveA1*, *SAV575*, *SAV576* and *SAV577*, and detection of *SAV575* transcription level in various *S. avermitilis* strains. *SAV577′*, 136-bp transcript from *SAV577* promoter region and the remainder ORF in D577. Error bars, standard deviations (*n* = 3). ***P<0.001 as determined by Student's *t*-test. ns, not significant. (**B**) Schematic representation of the relative positions of probes used for EMSAs. The lengths and positions of probes 1–7 were described previously [20]. Probe 577p, a 209-bp DNA fragment from positions −159 to −367 relative to the *SAV577* start codon, covering the putative transcriptional start point of *SAV577*. (**C**) EMSAs of the interaction of probes with purified His_6_-SAV577 protein. Each lane contained 0.3 nM labeled probe. (**D** and **E**) DNase I footprinting assay of SAV577 on the *SAV575* (**D**) and *SAV576* (**E**) promoter regions. The fluorograms represent control DNA (10 µM BSA) and protection reactions with increasing concentrations (0.08, 0.4, 0.8 µM) of His_6_-SAV577 protein.

*SAV577* is a *SAV576*-like gene and may therefore affect the expression of *SAV576* or related genes. To test this hypothesis, transcription of *ave* genes and *SAV577*-adjacent genes in ATCC31267 and D577 was determined by real-time RT-PCR. Transcription of *aveR* (which encodes a pathway-specific activator), *aveA1* (which encodes polyketide synthase AVES1), *SAV575*, and *SAV576* was upregulated in D577 relative to ATCC31267 (Fig. 4A). Deletion of *SAV577* did not cause notable alteration of its own transcription (*SAV577′*, *SAV577* promoter region and remainder ORF in D577). Taken together, these findings suggest that SAV577 affects avermectin biosynthesis by downregulating the transcription of *ave* genes and adjacent genes, *i.e.*, mimicking the effect of SAV576.

To further elucidate the regulatory role of *SAV577*, EMSAs were performed using full-length recombinant His_6_-SAV577. The probes designated 1–7 were described previously [20]. Probes 1–6 cover the entire *SAV575-SAV576* intergenic region, and probe 7 covers the 200-bp *aveR* promoter region (Fig. 4B). SAV577 bound to probes 1, 2, and 6, but not to probes 3, 4, 5, 7, or 577p (Fig. 4C). Probes 1 and 2 are located within the *SAV575* promoter region, and probe 6 within the *SAV576* promoter region [20]. The finding that SAV577 did not bind probe 577p within its own promoter region is consistent with the transcription analysis shown in Fig. 4A. The binding pattern of SAV577 was thus similar to that of SAV576 as reported previously [20], except for its own promoter. SAV577 is similar to SAV576 in that they both downregulate avermectin biosynthesis indirectly, repress *SAV575* transcription directly, and reciprocally repress each other's expression. However, SAV576 is autoregulated [20] whereas SAV577 is not.

DNase I footprinting assays were performed to determine the binding sites of SAV577 on the bidirectional *SAV575-SAV576* promoter region. SAV577 protected sites I, II, and III (Fig. 4D and 4E), the same as did SAV576 on this region [20]. This again indicated the similar regulatory mechanism of SAV576 and SAV577.

### SAV576 and SAV577 are Competitive Regulators

**Figure 5:**
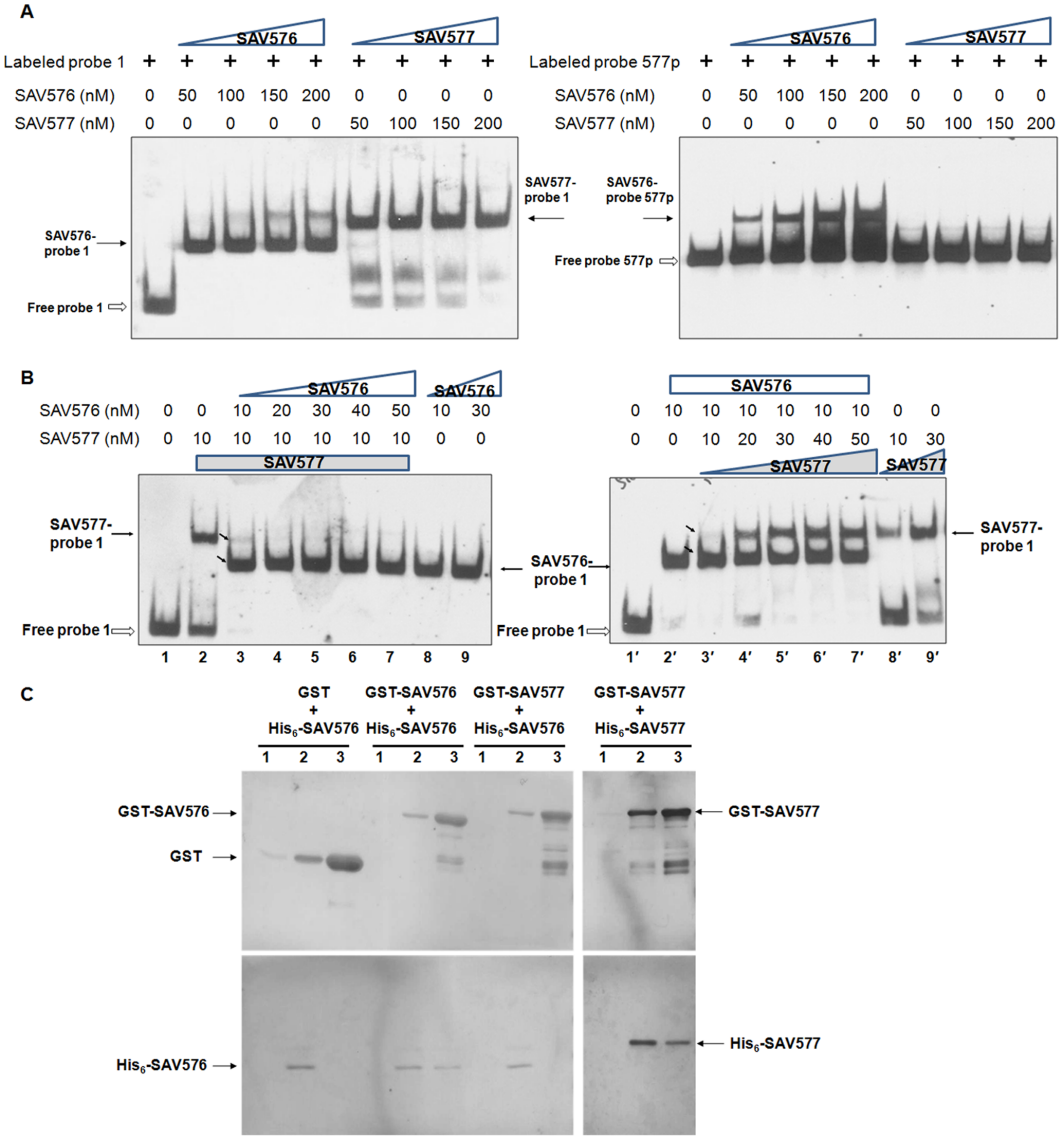
Relationships between SAV576 and SAV577. (**A**) Comparison of DNA-binding affinity of SAV576 and SAV577 with probes 1 and 577p. Each lane contained 0.15 nM labeled probe. White arrows: free probes. Black arrows: DNA-protein complexes. (**B**) Competitive EMSAs of probe 1 with His_6_-SAV576 and His_6_-SAV577 proteins. End-labeled probe 1 was incubated with the indicated concentrations of His_6_-SAV576 or His_6_-SAV577. Lanes 1 and 1′: control reactions (no protein added). Diagonal arrows: two complexes in lanes 3 and 3′. (**C**) GST pull-down assays of SAV576 and SAV577 from *E. coli* whole cell lysate. GST- and His_6_-tagged proteins were co-expressed in *E. coli*, lysed by sonication, and subjected to GST pull-down assay and Western blotting using anti-GST and anti-His antibodies, respectively. Lanes 1, cell lysate before IPTG induction. Lanes 2, cell lysate after induction. Lanes 3, GST pull-down.

The findings that SAV576 and SAV577 have the same binding sites on the *SAV575-SAV576* intergenic region and that SAV577 does not bind to its own promoter region suggest that these two proteins may compete for DNA binding and differ in their DNA-binding affinity. DNA-binding affinity of the two proteins was compared by incubating labeled probes 1 and 577p separately with various concentrations of His_6_-SAV576 or His_6_-SAV577 and performing EMSAs. Labeled probe 1 (0.15 nM) was retarded completely by 50 nM SAV576 but not by 200 nM SAV577; probe 577p was retarded by 50 nM SAV576 but not by 200 nM SAV577 (Fig. 5A). The DNA-binding affinity of SAV577 thus appears to be much weaker than that of SAV576.

To determine whether SAV576 and SAV577 compete for DNA binding, the two proteins were applied separately and together in EMSAs using probe 1. When applied separately, both proteins bound to probe 1 (Fig. 5B, lanes 2, 8, 9, 2′, 8′, 9′). In the presence of 10 nM His_6_-SAV577, increasing the concentration of His_6_-SAV576 resulted in the disappearance of SAV577-DNA complex and formation of SAV576-DNA complex (Fig. 5B, lanes 3-7). In the presence of 10 nM His_6_-SAV576, increasing the concentration of His_6_-SAV577 resulted in reduction of SAV576-DNA complex and formation of SAV577-DNA complex (Fig. 5B, lanes 3′-7′). When equimolecular 10 nM protein concentrations were used, the SAV576-DNA complex predominated over the SAV577-DNA complex (Fig. 5B, lanes 3 and 3′; diagonal arrows). These findings indicate that SAV576 and SAV577 compete for the same DNA region, and that DNA-binding affinity of SAV577 is weaker than that of SAV576.

To investigate the association between SAV576 and SAV577 under the physiological conditions within the bacterial cell, we co-expressed His_6_-SAV576 with GST, GST-576, or GST-577 in *E. coli*. GST pull-down experiments using whole cell lysates showed that His_6_-SAV576 was pulled down by GST-576 but not by GST or GST-577 (Fig. 5C, right). SAV576 thus interacts with itself *in vivo*, presumably to form homodimers like most TetR family transcriptional regulators, but does not interact with SAV577 directly. Similar formation of putative SAV577 homodimers was observed when His_6_-SAV577 and GST-SAV577 were co-expressed in *E. coli* (Fig. 5C, left).

### *SAV575* Transcription Levels in Various *S. avermitilis* Strains are Consistent with Avermectin Production Levels

*SAV575* is a cytochrome P450 family gene. Its product CYP102D1 was shown recently to catalyze the oxidation of saturated and unsaturated fatty acids with high regioselectivity [29]. We showed previously that *SAV575*, the important target gene of SAV576, has a promoting effect on avermectin production, and suggested that SAV575 functions to provide precursors for avermectin biosynthesis (*e.g.*, acetate and propionate extender units) by oxidizing fatty acids or other compounds [20]. Because *SAV575* is also a target gene of SAV577, we compared *SAV575* transcription levels in ATCC31267, D576, D577, and D576-577 (Fig. 4A). The *SAV575* transcription level was very low in ATCC31267 and was increased by deletion of *SAV576* or *SAV577*. The level was higher in D576 than that in D577, and highest in *SAV576-SAV577* double deletion mutant D576-577. The *SAV575* transcription levels in these strains were consistent with the avermectin production levels shown in Fig. 2A. These findings indicate that both SAV576 and SAV577 downregulate avermectin production primarily by repressing *SAV575* transcription.

## Discussion

The present study characterized the TFR SAV577 and demonstrated that it downregulates avermectin biosynthesis indirectly by a regulatory mechanism similar to that of SAV576. Deletion of *SAV576* or *SAV577* in *S. avermitilis* increased avermectin production, and the yield was further enhanced in *SAV576-SAV577* double deletion mutant D576-577. These findings suggest a strategy for improving industrial-scale avermectin production through deletion of *SAV576-SAV577* and overexpression of their target gene *SAV575*.

Two similar TFRs SAV576 and SAV577 had protective effects on the same sequence of the bidirectional *SAV575-SAV576* promoter region, but SAV576 displayed higher DNA-binding affinity. SAV577 has a stretch of 47 amino acid residues at its C-terminal region that are not present in SAV576; this difference may account for the weaker DNA-binding affinity of SAV577. SAV576 is autoregulated whereas SAV577 is not. The binding affinity of SAV576 to *SAV577* promoter region (probe 577p) was even low. Thus, SAV577 was unable to bind to its own promoter region probably because of its weaker DNA-binding affinity.

TFRs generally form homodimers that act as transcriptional regulators [14]. Despite the similarity of SAV577 to SAV576, each protein interacted with itself to form a putative homodimer, but that the two proteins did not interact with each other to form a heterodimer. Transcription levels of *SAV576* and *SAV577* were similar in wild-type ATCC31267, but the SAV576-DNA complex predominated over the SAV577-DNA complex for the same DNA region under equivalent protein concentrations. These findings suggest that SAV576 in *S. avermitilis* plays a dominant role in repressing target genes (including *SAV575*) and in inhibiting avermectin production. In the absence of SAV576, SAV577 exerts a similar inhibitory effect on avermectin production. Thus, the inhibitory effect of the SAV576 regulatory cascade on avermectin production was completely eliminated only when both SAV576 and SAV577 were absent, *i.e.*, in the double deletion mutant D576-577 (Fig. 2A). It is possible that *SAV576* and *SAV577* arose through a gene duplication event, such that one of them is able to regulate avermectin production (or other yet-unknown biological processes) in the absence of the other.

Although SAV577 and SAV576 do not directly regulate *aveR* transcription, *aveR* expression was upregulated by deletion of *SAV577* (Fig. 4A) or *SAV576* [20]. Possible explanations of these findings are that (i) an abundance of avermectin precursor resulting from increased *SAV575* transcription induces *aveR* expression; (ii) SAV576 and SAV577 control yet-unknown gene (s) that directly (or indirectly) regulate *aveR* expression. Studies to identify the direct transcriptional regulators of *aveR* in *S. avermitilis* are currently underway in our laboratory.

**Figure 6:**
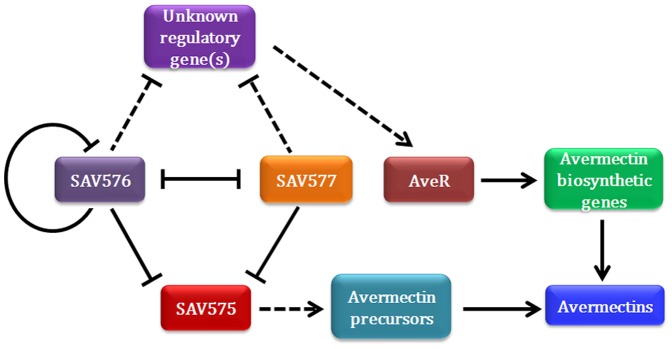
The possible regulatory pathway of SAV576/SAV577/SAV575 on avermectin biosynthesis in *S. avermitilis*. Two similar TFRs SAV576 and SAV577 both repress avermectin biosynthesis indirectly by (i) repressing *SAV575* expression directly, and SAV575 may provide precursors for avermectin biosynthesis; (ii) controlling unknown regulatory gene(s) that directly (or indirectly) regulate expression of the pathway-specific activator gene *aveR*, and AveR activates avermectin biosynthesis by activating the transcription of biosynthetic structural genes. SAV576 and SAV577 repress each other's expression. However, SAV576 is autoregulated whereas SAV577 is not. Arrows indicate activation, and bars indicate repression. Solid lines indicate direct control, and dotted lines indicate unknown routes.

Our findings clearly show that *SAV575* is an important target gene of SAV576 and SAV577 and is involved in avermectin production. A possible regulatory pathway of SAV576/SAV577/SAV575 on avermectin biosynthesis is thus proposed (Fig. 6). However, the possibility cannot be ruled out that other target genes also affect avermectin biosynthesis. Further experimental approaches such as ChIP-seq and bacterial one-hybrid systems will help identify other target genes of these two proteins. Increased understanding of the target genes and their functions will lead to more effective strategies for increasing industrial-scale avermectin production.

## Supporting Information

Figure S1 **Transcription levels of** ***SAV576*** **and** ***SAV577*** **in various** ***S. avermitilis*** **strains grown in FM-I medium for 10 days.** WT, wild-type strain ATCC31267. D576, *SAV576* deletion mutant. D577, *SAV577* deletion mutant. WT/pKC1139-577, *SAV577* overexpression strain. D576-577, *SAV576-SAV577* double deletion mutant. *hrdB* was used as an internal control. Each gene was examined by relative quantification real-time RT-PCR with gene-specific primers. Stars indicate no transcript. Standard deviations are indicated by error bars (n = 3). **P<0.01 and ***P<0.001 as determined by Student's *t*-test and denote that values reported are statistically significantly different between wild-type strain and mutant strains.
(TIF)

Table S1 **Primers used in this study.**
(DOC)

## References

[1] Challis GL, Hopwood DA (2003) Synergy and contingency as driving forces for the evolution of multiple secondary metabolite production by *Streptomyces* species. Proc Natl Acad Sci USA 100 Suppl 2 14555–14561. 12970466 10.1073/pnas.1934677100 PMC304118

[2] Demain AL (2002) Prescription for an ailing pharmaceutical industry. Nat Biotechnol 20: 331. 11923826 10.1038/nbt0402-331

[3] Watve MG, Tickoo R, Jog MM, Bhole BD (2001) How many antibiotics are produced by the genus *Streptomyces*? Arch Microbiol 176: 386–390. 11702082 10.1007/s002030100345

[4] Bibb MJ (2005) Regulation of secondary metabolism in *streptomycetes* . Current Opinion in Microbiology 8: 208–215. 15802254 10.1016/j.mib.2005.02.016

[5] Craney A, Ahmed S, Nodwell J (2013) Towards a new science of secondary metabolism. J Antibiot (Tokyo) 66: 387–400. 23612726 10.1038/ja.2013.25

[6] van Wezel GP, McDowall KJ (2011) The regulation of the secondary metabolism of *Streptomyces*: new links and experimental advances. Nat Prod Rep 28: 1311–1333. 21611665 10.1039/c1np00003a

[7] McCormick JR, Flardh K (2012) Signals and regulators that govern *Streptomyces* development. FEMS Microbiol Rev 36: 206–231. 22092088 10.1111/j.1574-6976.2011.00317.x PMC3285474

[8] Martin JF, Santos-Beneit F, Rodriguez-Garcia A, Sola-Landa A, Smith MC (2012) Transcriptomic studies of phosphate control of primary and secondary metabolism in *Streptomyces coelicolor* . Appl Microbiol Biotechnol 95: 61–75. 22622839 10.1007/s00253-012-4129-6

[9] Hesketh A, Chen WJ, Ryding J, Chang S, Bibb M (2007) The global role of ppGpp synthesis in morphological differentiation and antibiotic production in *Streptomyces coelicolor* A3(2). Genome Biol 8: R161. 17683547 10.1186/gb-2007-8-8-r161 PMC2374992

[10] Liu G, Chater KF, Chandra G, Niu G, Tan H (2013) Molecular regulation of antibiotic biosynthesis in *Streptomyces* . Microbiol Mol Biol Rev 77: 112–143. 23471619 10.1128/MMBR.00054-12 PMC3591988

[11] Burg RW, Miller BM, Baker EE, Birnbaum J, Currie SA (1979) Avermectins, new family of potent anthelmintic agents: producing organism and fermentation. Antimicrob Agents Chemother 15: 361–367. 464561 10.1128/aac.15.3.361 PMC352666

[12] Ikeda H, Omura S (1997) Avermectin Biosynthesis. Chem Rev 97: 2591–2610. 11851473 10.1021/cr960023p

[13] Ramos JL, Martinez-Bueno M, Molina-Henares AJ, Teran W, Watanabe K (2005) The TetR family of transcriptional repressors. Microbiol Mol Biol Rev 69: 326–356. 15944459 10.1128/MMBR.69.2.326-356.2005 PMC1197418

[14] Yu Z, Reichheld SE, Savchenko A, Parkinson J, Davidson AR (2010) A comprehensive analysis of structural and sequence conservation in the TetR family transcriptional regulators. J Mol Biol 400: 847–864. 20595046 10.1016/j.jmb.2010.05.062

[15] Ikeda H, Ishikawa J, Hanamoto A, Shinose M, Kikuchi H (2003) Complete genome sequence and comparative analysis of the industrial microorganism *Streptomyces avermitilis* . Nat Biotechnol 21: 526–531. 12692562 10.1038/nbt820

[16] Duong CT, Lee HN, Choi SS, Lee SY, Kim ES (2009) Functional expression of *SAV3818*, a putative TetR-family transcriptional regulatory gene from *Streptomyces avermitilis*, stimulates antibiotic production in *Streptomyces* species. J Microbiol Biotechnol 19: 136–139. 19307761 10.4014/jmb.0806.387

[17] Miyamoto KT, Kitani S, Komatsu M, Ikeda H, Nihira T (2011) The autoregulator receptor homologue AvaR3 plays a regulatory role in antibiotic production, mycelial aggregation and colony development of *Streptomyces avermitilis* . Microbiology 157: 2266–2275. 21622528 10.1099/mic.0.048371-0

[18] He F, Liu W, Sun D, Luo S, Chen Z (2014) Engineering of the TetR family transcriptional regulator SAV151 and its target genes increases avermectin production in *Streptomyces avermitilis* . Appl Microbiol Biotechnol 98: 399–409. 24220792 10.1007/s00253-013-5348-1

[19] Liu Y, Yan T, Jiang L, Wen Y, Song Y (2013) Characterization of SAV7471, a TetR-family transcriptional regulator involved in the regulation of coenzyme A metabolism in *Streptomyces avermitilis* . J Bacteriol 195: 4365–4372. 23893108 10.1128/JB.00716-13 PMC3807474

[20] Guo J, Zhang X, Luo S, He F, Chen Z (2013) A novel TetR family transcriptional regulator, SAV576, negatively controls avermectin biosynthesis in *Streptomyces avermitilis* . PLoS One 8: e71330. 23967193 10.1371/journal.pone.0071330 PMC3742746

[21] Ikeda H, Kotaki H, Tanaka H, Omura S (1988) Involvement of glucose catabolism in avermectin production by *Streptomyces avermitilis* . Antimicrob Agents Chemother 32: 282–284. 3364948 10.1128/aac.32.2.282 PMC172155

[22] Macneil DJ, Klapko LM (1987) Transformation of *Streptomyces avermitilis* by plasmid DNA. J Indust Microbiol 2: 209–218.

[23] Kieser T, Bibb MJ, Buttner MJ, Chater KF, Hopwood DA (2000) Practical *Streptomyces* genetics: a laboratory manual. Norwich: John Innes Foundation.

[24] Chen Z, Wen J, Song Y, Wen Y, Li JL (2007) Enhancement and selective production of avermectin B by recombinants of *Streptomyces avermitilis* via intraspecific protoplast fusion. Chinese Science Bulletin 52: 616–622.

[25] Guo J, Zhao JL, Li LL, Chen Z, Wen Y (2010) The pathway-specific regulator AveR from *Streptomyces avermitilis* positively regulates avermectin production while it negatively affects oligomycin biosynthesis. Molecular Genetics and Genomics 283: 123–133. 20012992 10.1007/s00438-009-0502-2

[26] Bierman M, Logan R, Obrien K, Seno ET, Rao RN (1992) Plasmid cloning vectors for the conjugal transfer of DNA from *Escherichia coli* to *Streptomyces* spp. Gene 116: 43–49. 1628843 10.1016/0378-1119(92)90627-2

[27] Zhao JL, Wen Y, Chen Z, Song Y, Li JL (2007) An *adpA* homologue in *Streptomyces avermitilis* is involved in regulation of morphogenesis and melanogenesis. Chinese Science Bulletin 52: 623–630.

[28] Zianni M, Tessanne K, Merighi M, Laguna R, Tabita FR (2006) Identification of the DNA bases of a DNase I footprint by the use of dye primer sequencing on an automated capillary DNA analysis instrument. J Biomol Tech 17: 103–113. 16741237 PMC2291779

[29] Choi KY, Jung E, Jung DH, Pandey BP, Yun H (2012) Cloning, expression, and characterization of CYP102D1, a self-sufficient P450 monooxygenase from *Streptomyces avermitilis* . FEBS J 279: 1650–1662. 22188665 10.1111/j.1742-4658.2011.08462.x

