# Supplementary figures and images for: Two Adjacent and Similar TetR Family Transcriptional Regulator Genes, SAV577 and SAV576, Co-Regulate Avermectin Production in Streptomyces avermitilis
Source: PLoS One. 2014 Jun 10;9(6):e99224. doi: 10.1371/journal.pone.0099224 (PMC4051647; doi:10.1371/journal.pone.0099224)

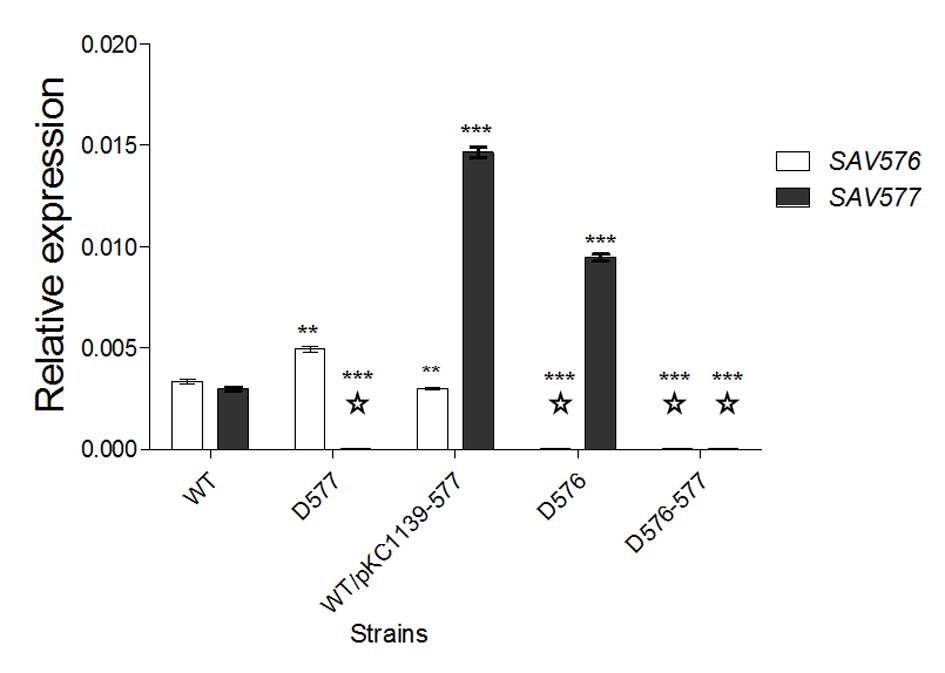

Supplement: Figure S1 — Transcription levels of SAV576 and SAV577 in various S. avermitilis strains grown in FM-I medium for 10 days. WT, wild-type strain ATCC31267. D576, SAV576 deletion mutant. D577, SAV577 deletion mutant. WT/pKC1139-577, SAV577 overexpression strain. D576-577, SAV576-SAV577 double deletion mutant. hrdB was used as an internal control. Each gene was examined by relative quantification real-time RT-PCR with gene-specific primers. Stars indicate no transcript. Standard deviations are indicated by error bars (n = 3). **P<0.01 and ***P<0.001 as determined by Student's t-test and denote that values reported are statistically significantly different between wild-type strain and mutant strains. (TIF) [file pone.0099224.s001.tif]
