# Supplementary material for: Two Adjacent and Similar TetR Family Transcriptional Regulator Genes, SAV577 and SAV576, Co-Regulate Avermectin Production in Streptomyces avermitilis
Source: PLoS One. 2014 Jun 10;9(6):e99224. doi: 10.1371/journal.pone.0099224 (PMC4051647; doi:10.1371/journal.pone.0099224)
Supplement: Table S1 — Primers used in this study. (DOC) [file pone.0099224.s002.doc]

**Table S1. Primers used in this study.**

| Primer | | | DNA sequence (5'-3') | | Use |
| --- | --- | --- | --- | --- | --- |
| Primers for gene disruption, complementation, and overexpression | | | | | |
| GJ147 | CGGAATTCCGCAACGCTTCACAACTC, *Eco*RI | | | | 5′ flanking region of *SAV577* |
| GJ148 | GAAGATCTTACGCTTCGCTCTTTCCATAC, *Bgl*II | | | |
| GJ149 | GAAGATCTGAAGTTCAGTGAGGGCCAAA, *Bgl*II | | | | 3′ flanking region of *SAV577* |
| GJ150 | CCCAAGCTTGTGTGTTCACGGGTCTGCT, *Hin*dIII | | | |
| GJ151 | GGGATGTGATGGGCG | | | | Confirm *SAV577*-deleted mutant D577 |
| GJ152 | TGCCCGACGAGGAGAT | | | |
| GJ87 | GCCGAACAAGGAAGCATTG | | | |
| GJ88 | ATCGTGTCCAGCACCAGG | | | |
| GJ151 | GGGATGTGATGGGCG | | | | Confirm double-deleted mutant D576-577 |
| GJ152+ | GTATGGCGACTGAACGGAAG | | | |
| GJ87 | GCCGAACAAGGAAGCATTG | | | |
| GJ88 | ATCGTGTCCAGCACCAGG | | | |
| GJ223 | CGGAATTCCCGACATCACCACCCTGT, *Eco*RI | | | | Complement D577 and overexpress *SAV577* |
| GJ224 | GCTCTAGAGGCACCTTGTTATGCAGTCC, *Xba*I | | | |
| GJ229 | AACCATATGGAAAGAGCGAAGCGT, *Nde*I | | | | For His_6_-tagged SAV577 protein |
| GJ230 | GCTGAATTCGTCCAGTGCGCCTCAAGG, *Eco*RI | | | |
| GJ231 | CCGGAATTCATGGCGACTGAACGGAAG, *Eco*RI | | | | For GST-tagged SAV576 protein |
| GJ232 | CCGCTCGAGTCGGTGACACAGGACAGG, *Xho*I | | | |
| GJ233 | CCGGAATTCATGGAAAGAGCGAAGCGTAC, *Eco*RI | | | | For GST-tagged SAV577 protein |
| GJ234 | ATTTGCGGCCGCAACTACAGGCACGGCTGAAC, *Not*I | | | |
| Primers for ChIP assay | | | | | |
| GJ113 | GCTCTCGGAATTGTTCGTTC | | | Detect *SAV577* promoter DNA | |
| GJ114 | CGTTCGTCCACTGATACTTTC | | |
| B1n | ACCCTCGGATCGTGC | | | Detect *hrdB* promoter DNA | |
| B2n | AGCGCCATGACAGAGAC | | |
| Primers for real-time RT-PCR | | | | | |
| GJ93 | ATCTGCACCCCTTCCTCAAC | | | *SAV575* ORF | |
| GJ94 | ACTTCTTGTCATCGGCCTTG | | |
| GJ137 | GGGCACAAGAAAGAGAGACG | | | *SAV576* ORF | |
| GJ138 | CGTGGTGGTACAGCGACAT | | |
| GJ177 | CCGATGGTCCGCTCTATTT | | | *SAV577′*, *SAV577* non-coding region | |
| GJ178+ | CGTACGGCGTATGGAAATCT | | |
| GJ87+ | TACTCGGAAGAGGGATGTGC | | | *SAV577* ORF | |
| GJ88+ | GAGTTTGCGCATCGTGAGT | | |  | |
| GJ97 | CAGAAGAACTCACGCTCGTC | | | *aveR* ORF | |
| GJ98 | ACTCTTTCCACAGCCCATTC | | |
| GJ99 | CGGACAGGACTACGCACTTC | | | *aveA1* ORF | |
| GJ100 | ACGAGATACGACCGGAGATG | | |
| GJ91 | CCAAGGGCTACAAGTTCTCC | | | *hrdB* ORF | |
| GJ92 | TTGATGACCTCGACCATGTG | | |
| Primers for EMSA | | | | | |
| GJ113 | | GCTCTCGGAATTGTTCGTTC | | | Probe 577p |
| GJ114 | | CGTTCGTCCACTGATACTTTC | | |
| hrdBP11 | | CATCGTTGACCACCTATGACC | | | *hrdB* promoter |
| hrdBP22 | | CTCTCGGAACGTTGGAAAAC | | |
| GJ209 | | GAAGGCATACAGCGTATGGAAAC | | | Probe 1 |
| GJ210 | | ATGGCTGGCTCCTCCAAG | | |
| GJ123 | | CCCAGGTCTGCCGATTGAGTTTCCATACGTCGTATGGTACTAC | | | Probe 2 |
| GJ124 | | GTAGTACCATACGACGTATGGAAACTCAATCGGCAGACCTGGG | | |
| GJ141 | | ACCTCCGCGATGCGTACT | | | Probe 3 |
| GJ142 | | GAAACTCAATCGGCAGACCT | | |
| GJ181 | | ATATGCCCTGCCGAGGAG | | | Probe 4 |
| GJ182 | | GGAGGACACCCACGATCC | | |
| GJ139 | | AGGACGGTGGCATCGTTT | | | Probe 5 |
| GJ140 | | GTTCGCCAGGGTCTCCTC | | |
| GJ219 | | AGTCGCCATACGCTGTACGGTACCAGCGCCCGCGGCACTGCGG | | | Probe 6 |
| GJ220 | | CCGCAGTGCCGCGGGCGCTGGTACCGTACAGCGTATGGCGACT | | |
| GJ105 | | GGTATTCCATTCGGTGTTGC | | | Probe 7 |
| GJ106 | | TGTTATGAATTTGCCCTGGTG | | |
| Primers for DNase I footprinting | | | | | |
| GJ77 | GAGACCCTGGCGAACC | | | | *SAV575- SAV576*  intergenetic region |
| FAM-GJ78 | GGCTGGCTCCTCCAAG | | | |
| GJ227 | CAGGGCATATCTGCGATCC | | | |
| FAM-GJ228 | AGTTTGCGCATGGTGAGC | | | |
